# Sternoclavicular Joint Tracheal Fistula: An Unusual Postradiation Complication in a Laryngectomee

**DOI:** 10.1155/crot/8268690

**Published:** 2025-05-30

**Authors:** Elena Dina, Beatriz Pallarés Martí, Vincenzo Filomena, Mario Prenafeta Moreno, Juan José Díaz Argüello, Carmen María Blázquez Mañá, Joël Sánchez Fernández, Yolanda Escamilla Carpintero

**Affiliations:** ^1^Otorhinolaryngology Department, Parc Tauli Health Corporation Consortium, Parc Tauli 1, Sabadell 08208, Spain; ^2^Neuroradiology Division, Diagnostic Imaging Department, Parc Tauli Health Corporation Consortium, Parc Tauli 1, Sabadell 08208, Spain; ^3^Pathology Department, Parc Tauli Health Corporation Consortium, Parc Tauli 1, Sabadell 08208, Spain; ^4^Orthopedic and Trauma Surgery Department, Parc Tauli Health Corporation Consortium, Parc Tauli 1, Sabadell 08208, Spain; ^5^Oncology Division, Otorhinolaryngology Department, Parc Tauli Health Corporation Consortium, Parc Tauli 1, Sabadell 08208, Spain

**Keywords:** clavicular osteomyelitis, clavicular osteoradionecrosis, sternoclavicular joint fistula, sternoclavicular joint osteomyelitis, sternoclavicular joint osteoradionecrosis

## Abstract

A 68-year-old man previously treated for a large laryngeal neoplasm (pT4 pN0 squamous cell carcinoma) developed osteomyelitis of the medial third of the right clavicle with the formation of a fistula between the sternoclavicular joint and tracheal wall near the tracheostomy border. The clinical course was tedious, required prolonged antibiotic trials, and extended surgical bone resection to control the infection. The final outcome was favorable with wound closure although the patient was left with permanent limitation of shoulder abduction (his shoulder mobility had been normal prior to this process). Histopathological examination of the resected bone suggested a diagnosis of both osteoradionecrosis and osteomyelitis. Indeed, differential diagnosis between these two entities can be challenging after radiotherapy. Here, we present a review of the relevant academic literature and discuss the therapeutic options.

## 1. Introduction

Sternoclavicular joint (SCJ) complications in head and neck cancer patients following radiation therapy are extremely rare. Nonetheless, several cases of SCJ osteomyelitis (OSM) and SCJ osteoradionecrosis (ORN) have been reported for this group. Almost all SCJ ORN patients had a prolonged local infection and more than half of the cases of SCJ OSM in head and neck patients had previously been treated with radiation therapy [1–4].

We report the case of a patient who had previously undergone radiation therapy that subsequently presented a fistula between the right SCJ and trachea, near the tracheostomy border. The symptoms and the radiological appearance of the lesion were suggestive of OSM but the histopathology slides showed an overlap of ORN and OSM criteria. In this case, late tissue injury from radiation treatment had caused the ulceration of the tracheostomy border and the adjacent tracheal wall, which had then facilitated a local infection and prevented the normal reaction of the bone tissue, which would have otherwise contained it.

The incidence of ORN is 4%–22% in head and neck cancer, the mandible being the most affected bone and oral cancer the most affected subtype. Clavicle ORN is a well-documented complication in cases of breast or lung malignancy. The time of onset of the ORN varies from 2 days up to 13 years after completion of radiotherapy, with a mean range of 6 months and most cases debuting in the first year. Moreover, the outcome is not affected by the time passed since the end of the radiation treatment and the onset of the symptomatology [5]. In turn, SCJ OSM can occur up to 5 years posttreatment in head and neck cancer cases. Complications of clavicular OSM include hemorrhage of the great vessels, pathologic fractures, mediastinitis, sepsis, and death [1, 3].

## 2. Case Presentation

This was the case of a 68-year-old man with personal antecedents of smoking 10 cigarettes a day for 50 years, HLA-B27–positive ankylosing spondylitis, a cardiac pacemaker, and benign prostate hypertrophy. The patient had been diagnosed with a large squamous cell carcinoma of the larynx (stage was cT4cN2c/pT4a pN0), which had been treated by total laryngectomy, bilateral functional neck dissection, central neck dissection, and insertion of a voice prosthesis.

Adjuvant radiotherapy with 60 Gy on the tumor site and 54 Gy on the Cervical areas II, III, IV, and VI had also been carried out. According to the Common Toxicity Criteria of Adverse Events, at the end of the radiation therapy, he presented xerostomia (Grade 1), odynophagia (Grade 2), and peristomal skin reaction (Grade 2 with Grade 3 foci). The acute radiation had induced peristomal dermatitis, which required prolonged wound care with hydrocolloid dressings.

**Figure 1:**
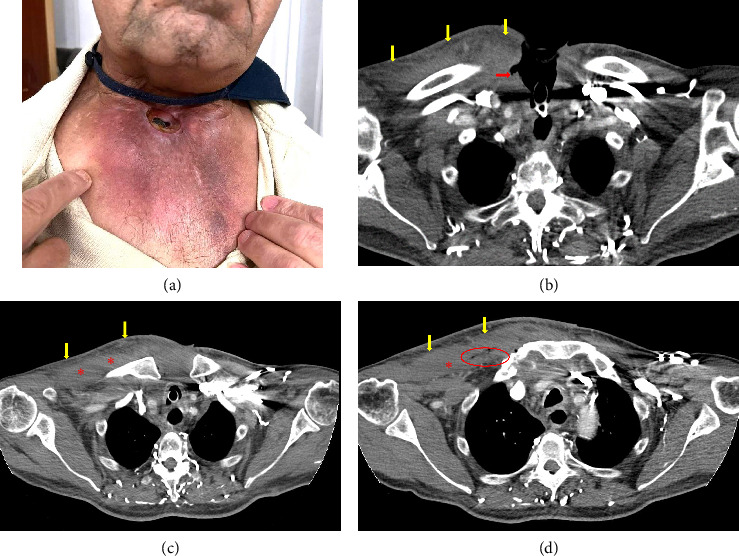
(a) Initial case presentation: right SCJ and adjacent anterior chest wall cellulitis. First axial CT scan slides (b). The red arrow shows focal ulceration of the right side of the tracheostomy; the yellow arrows show soft tissue inflammatory changes (cellulitis). (c and d) Chest wall inflammatory changes (cellulitis) and right pectoral major myositis (asterisk) with gas formation (red circle).

Four months after completion of the radiotherapy, the patient came to the emergency department with sternoclavicular pain and cellulitis of the right anterior chest wall. A computed tomography (CT) scan and blood test suggested cellulitis of the pectoralis major with gas formation and a loss of cutaneous continuity on the right tracheostoma border. Empiric antibiotic treatment was started with amoxicillin–clavulanic acid. The initial culture of an ultrasound-guided fine needle aspiration (FNA) of the right anterior chest wall was negative (Figure 1).

**Figure 2:**
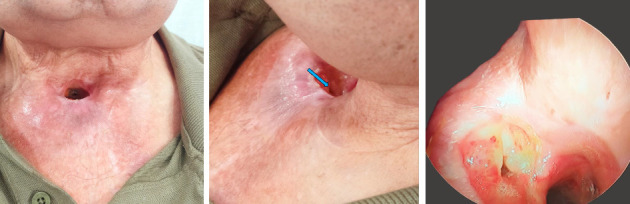
Front and oblique view: better infection control, the blue arrow shows SCJ–tracheal fistula. Weeks later: a 30° endoscope view of the fistula with articular liquid and air bubbles.

Three weeks later, after local worsening, a second ultrasound-guided FNA of the right infraclavicular area was taken, and the culture was positive for methicillin-sensitive *Staphylococcus aureus*, leading to a treatment switch to piperacillin–tazobactam. Nevertheless, the infection continued to progress and a fistula between the right SCJ and trachea appeared near the tracheostomy border (Figure 2 and Supporting videos 1 and 2).

**Figure 3:**
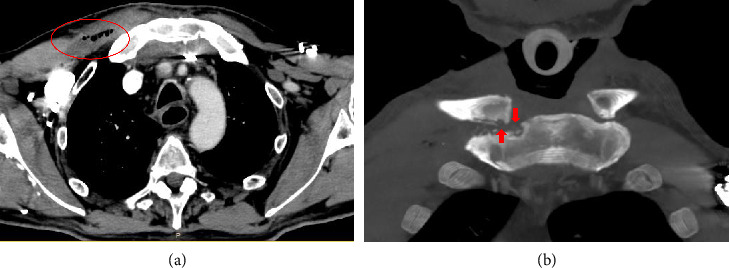
CT scan at 3 weeks: (a) liquid collection containing gas (red circle) in the right pectoralis major. (b) Both clavicle and sternum bone erosions (red arrows).

**Figure 4:**
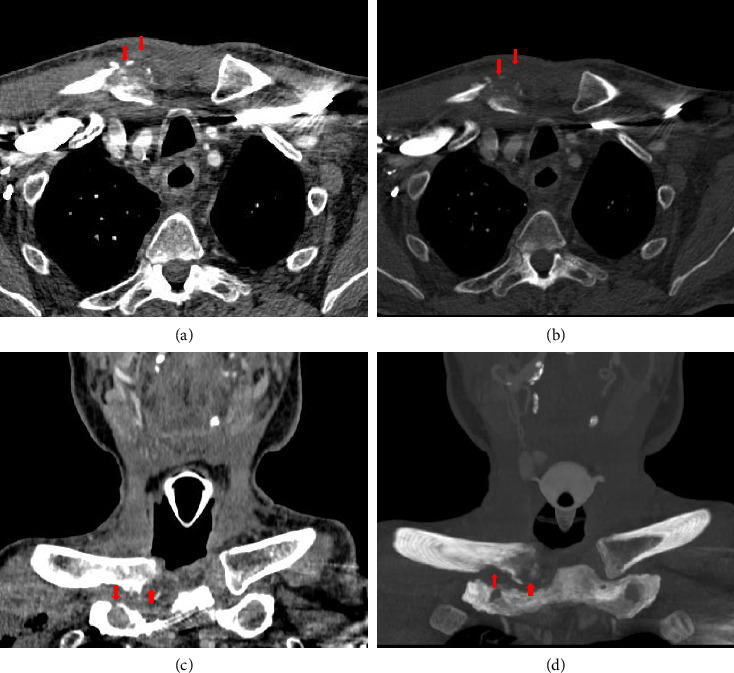
CT scan at 6 weeks later after clinical worsening: (a and b) axial and (c and d) coronal slides. Signs of sternoclavicular joint arthritis with enlargement of the bone erosions, osteolysis, and soft tissue augmentation (red arrows).

A biopsy of the perifistulous granulation tissue was negative for malignancy. Furthermore, CT scans at 3 and 6 weeks after the appearance of this lesion showed the presence of bone erosions on both the clavicle and sternum, osteolysis, and increased soft tissue in the area (Figures 3 and 4).

After completing 12 weeks of piperacillin–tazobactam (4 g/0.5 g every 8 h intravenous), resection of the medial portion of the clavicle and local reconstruction with a major pectoralis rotation flap were planned. The surgery was postponed because of a relapse in the *S. aureus* infection which was treated with cefazolin (1 g every 8 h, intravenous, for 3 weeks). Then, the surgical procedure was performed. The patient was treated perioperatively with daptomycin (10 mg/kg/day) and meropenem (1 g/8 h intravenous) for 3 days.

The fistulous tract between the SCJ and trachea had already closed spontaneously prior to surgery (Supporting video 3).

**Figure 5:**
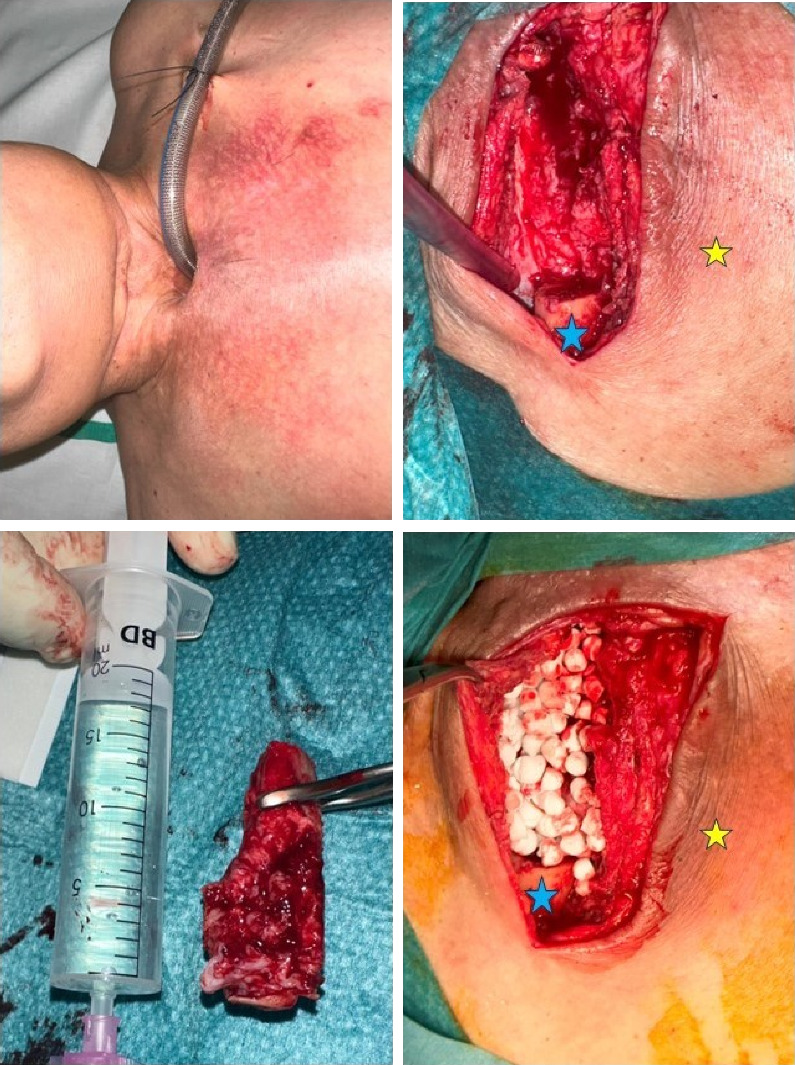
Resection of the medial third of the clavicle, a fibrous capsule closed the fistula tract, and the space was filled with STIMULAN rapid cure beads soaked in vancomycin and gentamicin; blue star: lateral portion of the right clavicle; yellow star: right subclavicular region.

During the operation, resection of the medial third of the clavicle was performed, and vigorous lavage of the sternoclavicular capsule ruled out communication with the trachea (using a Pulsavac Plus AC Wound Debridement System, Zimmer Biomet). Therefore, the surgical plan was revised and the defect was filled with STIMULAN rapid cure beads (Biocomposites) soaked in vancomycin and gentamicin, and the wound was closed primarily without the need of a reconstruction flap (Figure 5).

**Figure 6:**
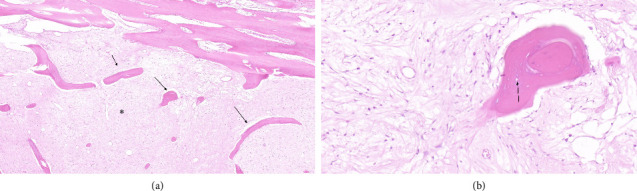
(a) Hematoxylin–eosin (HE) stained, ×10 magnification: ORN with dense fibrosis (^∗^), chronic inflammatory infiltrate (OSM), and empty bone trabeculae (arrows). (b) HE stained, ×20 magnification: the image of a residual bone trabecula with osteonecrosis and clusters of empty osteocytes without viable nuclei (arrow).

The result of the histopathological analysis of the specimen was consistent with both ORN and OSM, with the presence of dense fibrosis, residual bone trabeculae, osteocytes without viable nuclei, and abundant inflammatory infiltrates (Figures 6(a) and 6(b)). Moreover, *S. aureus* methicillin-sensitive colonies grew on the culture samples of the resected bone.

**Figure 7:**
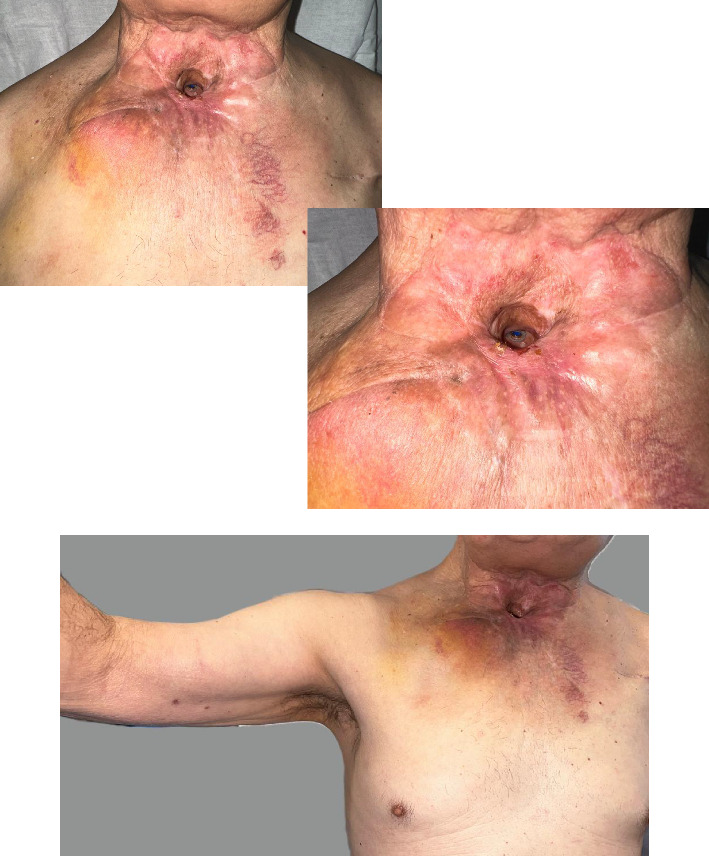
Postoperative at 1 month: local healing and limited arm abduction.

**Figure 8:**
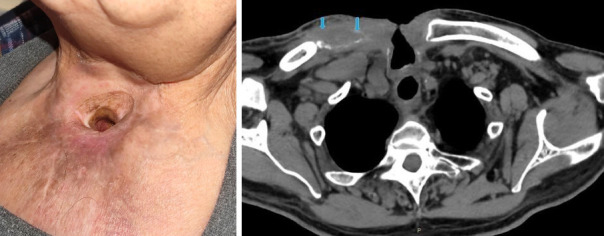
Postoperative at 6 months: clinical stability. Axial CT scan: fibrosis filling the clavicular resection space (blue arrows).

Postoperatively, the patient was treated with levofloxacin (750 mg daily) for 16 weeks. He healed very well, although the abduction of the shoulder was limited, and there was some residual pain controllable with conventional analgesia (Figures 7 and 8).

The whole process took over 9 months and the patient remained stable for at least 6 months after the end of the last antibiotic treatment.

## 3. Discussion

Long-term postirradiation periclavicular complications include infections, ORN, metastatic tumors, postirradiation sarcoma, pathologic fractures, major hemorrhage, and processes unrelated to primary cancer. Thus, due to the clinical similarities between some of these scenarios, a local biopsy is fundamental for a differential diagnosis [6–8].

Of note, SCJ OSM has been reported in less than 20 cases of head and neck cancer to date. More than half of these patients received previous radiation therapy for their primary malignancy, making this the most important risk factor for SCJ OSM [1–3, 6].

Several cases of clavicular ORN with SCJ involvement in head and neck cancer patients have been previously described in the academic literature. However, to the best of our knowledge, only one has described the formation of a fistula between the SCJ and trachea in a patient with a primary pyriform sinus squamous cell carcinoma treated with radiotherapy [4, 5, 9]. The presentation of bilateral clavicular and SCJ ORN with peristomal wounds in a patient with tongue squamous cell carcinoma was also described [10].

The incidence of ORN in cases of head and neck for laryngopharynx cancer is 4.1%. Advanced tumor stage, age, radiation technique, total dose, local trauma, immune defects, malnutrition, and infection all increase the rates of ORN. Furthermore, the appearance of this complication has significantly decreased with advances in radiotherapy, especially intensity-modulated therapy [11].

ORN is characterized by three evolutive stages. The first is the preproliferative phase, with acute inflammation and endothelial cell changes. The second is the constitutive organized phase in which there is intense fibroblastic activity and extracellular matrix disorganization. The third is the fibroatrophic phase marked by progressive osteoclast resorption, the presence of empty bone lacunae, osteocyte lysis (which is pathognomonic for ORN), extensive demineralization, and finally, myofibroblast apoptosis. Nonetheless, differential diagnosis between ORN and OSM can be challenging. A diagnosis of ORN usually requires a negative bone specimen culture (bacterial colonization vs. infection per se in cases of OSM). However, infection may still occur in debilitated irradiated bone, and so both conditions can sometimes overlap [12–14].

In this present case, postradiation peristomal ulceration allowed the infection, and local chronic ischemic changes favored its persistence. The histopathology slides were more suggestive of ORN because microorganisms and reactive viable bone formation were not observed. In turn, an inflammatory infiltrate is usually present in both entities, albeit at a higher density in cases of OSM. Although the lateral portion of the clavicle had a normal appearance on the CT scans, the clinical evolution and the presence of the SCJ–tracheal fistula for months, alongside the chronic infection, suggested that both ORN and OSM were present in this case.

A case of septic arthritis of the SCJ has recently been published in a tracheostomized patient without prior radiation exposure [15]. This case shows that radiotherapy is not essential for the development of OSM, but the ORN associated in our case worsens and prolongs the evolution, overlapping two different conditions, thus posing complex clinical challenges.

There is some evidence for hyperbaric oxygen as an adjuvant option in both ORN and OSM [16], but it was not used in this particular case due to logistical issues.

The correct diagnosis and management of clavicular and SCJ ORN, as well as OSM, require a multidisciplinary team involving infection control, extended surgical resection, and local closure of tissue defects with locoregional flaps or microvascular-free tissue transfer.

## Data Availability

All data accessible are included in this paper and no new data are generated.

## Supporting Information

Supporting videos 1 and 2: SCJ–tracheal fistula with granulation tissue and a small leak of the articular liquid into the trachea, 30° endoscope view. Supporting video 3: Preoperative appearance: SCJ–tracheal fistula with granulation tissue and without an articular liquid leak into the trachea, and limited right arm abduction.
